# Coffee Consumption and Blood Pressure: Results of the Second Wave of the Cognition of Older People, Education, Recreational Activities, Nutrition, Comorbidities, and Functional Capacity Studies (COPERNICUS)

**DOI:** 10.3390/nu13103372

**Published:** 2021-09-25

**Authors:** Agnieszka Kujawska, Sławomir Kujawski, Weronika Hajec, Natalia Skierkowska, Małgorzata Kwiatkowska, Jakub Husejko, Julia L. Newton, Jose Augusto Simoes, Paweł Zalewski, Kornelia Kędziora-Kornatowska

**Affiliations:** 1 Department of Physiology, Collegium Medicum in Bydgoszcz, Nicolaus Copernicus University in Toruń, 85-092 Bydgoszcz, Poland; 2 Department of Exercise Physiology and Functional Anatomy, Collegium Medicum in Bydgoszcz, Nicolaus Copernicus University in Torun, 85-094 Bydgoszcz, Poland; skujawski@cm.umk.pl (S.K.); p.zalewski@cm.umk.pl (P.Z.); 3 Department of Geriatrics, Collegium Medicum in Bydgoszcz, Nicolaus Copernicus University in Toruń, 85-094 Bydgoszcz, Poland; weronika.topka.bydg@gmail.com (W.H.); nataliaskierkowska1@gmail.com (N.S.); malgorzata.gajos0904@gmail.com (M.K.); kubahusejko@gmail.com (J.H.); kasiakor@interia.pl (K.K.-K.); 4 Population Health Sciences Institute, The Medical School, Newcastle University, Newcastle-upon-Tyne NE2 4AX, UK; julia.newton@ncl.ac.uk; 5 Department of Medical Sciences, University of Beira Interior, 6200-506 Covilha, Portugal; jars@ubi.pt

**Keywords:** hypertension, coffee, Poland, longitudinal study

## Abstract

This study examined the relationship between the frequency of coffee consumption and blood pressure over a two year follow up of a cohort of elderly people. Healthy, older people (N = 205) were examined at baseline and at two years. Participants completed physical and behavioural assessments, which included body composition, current pharmacological treatment, and frequency of coffee consumption grouped into three categories: “never to a few times per month”, “once a week to a few times per week”, and “every day”. Blood pressure (systolic (sBP), diastolic (dBP), mean (mBP), and pulse pressure (PP)) was measured at baseline and after two years. After adjusting for body composition, smoking status, age, sex, heart rate, and number of antihypertensive agents taken, participants who drank coffee everyday had a significant increase in sBP, with a mean of 8.63 (1.27; 15.77) and an mBP, with a mean of 5.55 mmHg (0.52; 10.37) after two years (t = 2.37, *p* = 0.02 and t = 2.17, *p* = 0.03, respectively) compared to participants who never or very rarely (up to a few times per month) drank coffee. DBP and PP were not affected by coffee consumption frequency in a statistically significant manner.

## 1. Introduction

### 1.1. Hypertension

The aetiology of hypertension is unclear in most cases, but is associated with multiple risk factors [1]. Prevalence of hypertension is higher (two times more) in older compared to younger populations [2]. Aging, which is a time-associated decline in tissue functionality, increases the risk of developing metabolic syndromes. Aged-related changes, such as chronic inflammation, endothelial dysfunction, a decrease in elastin, an increase in collagen and calcification of arteries, an increase in sympathetic nervous system activity, and an increase in aldosterone production and salt sensitivity, are potential factors that could increase the occurrence of hypertension [3]. Several studies showed that aging thickens the walls of large conduit arteries, thereby decreasing their elasticity. Higher arterial stiffness reduces the compliance of arteries in close proximity to the heart, leading to higher systolic and pulse pressure. Therefore, isolated systolic hypertension might be a result of age-related cardiovascular alterations [2].

### 1.2. Coffee

Coffee is a widely consumed pharmacologically active beverage. Coffee consumption in the European community is similar to that in the United States, which is estimated to be 5.1 kg/year per person [4]. Coffee contains caffeine and thousands of other chemical compounds, including lipids, carbohydrates, nitrogenous, minerals, vitamins, alkaloids, and phenolic compounds [5].

### 1.3. Acute Effects of Coffee Consumption on BP

Coffee ingestion has an acute effect by increasing blood pressure. Ingestion of two to three cups of coffee increases systolic blood pressure (sBP) by 3–14 mmHg and diastolic blood pressure (dBP) by 4–13 mmHg [6]. The acute pressor effect of coffee might be more pronounced in those who are not habitual coffee drinkers [6]. The blood pressure dysregulations, which were observed after acute coffee intake, were attributed to caffeine. Studies have demonstrated that caffeine promotes sympathetic antagonism of adenosine receptor, norepinephrine release via direct effects on the adrenal medulla, activation of the renin angiotensin renal system, and might be the mechanism of coffee-related acute hypertension [7]. In contrast, the benefits of caffeine ingestion in young healthy men were also reported. Acute administration of caffeine led to improvement in endothelial vasodilatory function [8]. Nevertheless, animal model studies showed reduced catecholamines circulating levels with chronic caffeine consumption [9]. Importantly, there is a remarkable and fast blood pressure adaptation to coffee consumption, which appears just a few days after the first intake and attenuates the coffee-related acute increase in blood pressure [10,11].

### 1.4. Chronic Effects of Coffee Consumption on BP

Currently, coffee consumption is not considered a risk factor for hypertension development. However, results derived from studies of chronic coffee consumption and its effect on blood pressure are ambiguous. Higher coffee intake was associated with a lower risk of developing hypertension [12]. However, high coffee consumption was also related to high blood pressure, but this effect could be smoking-dependent [12,13]. A systematic meta-analysis concluded that routine coffee consumption of more than three cups per day was not related to a higher risk of hypertension when compared to low consumption (less than one cup per day) [14].

The aim of our study was to examine the relationship between the frequency of coffee consumption and changes in blood pressure over a two year follow up period in an elderly cohort.

## 2. Materials and Methods

### 2.1. Study Group

#### 2.1.1. Sample Size Calculation

The sample size calculation was conducted using the general linear mixed model power and sample size 3.0 calculator (GLIMMPSE 3.0) [15]. The study presented here was part of a larger study, for which the primary outcome was the score change in cognitive function. The sample size was, therefore, calculated as described previously [16].

#### 2.1.2. Study Recruitment

**Figure 1:**
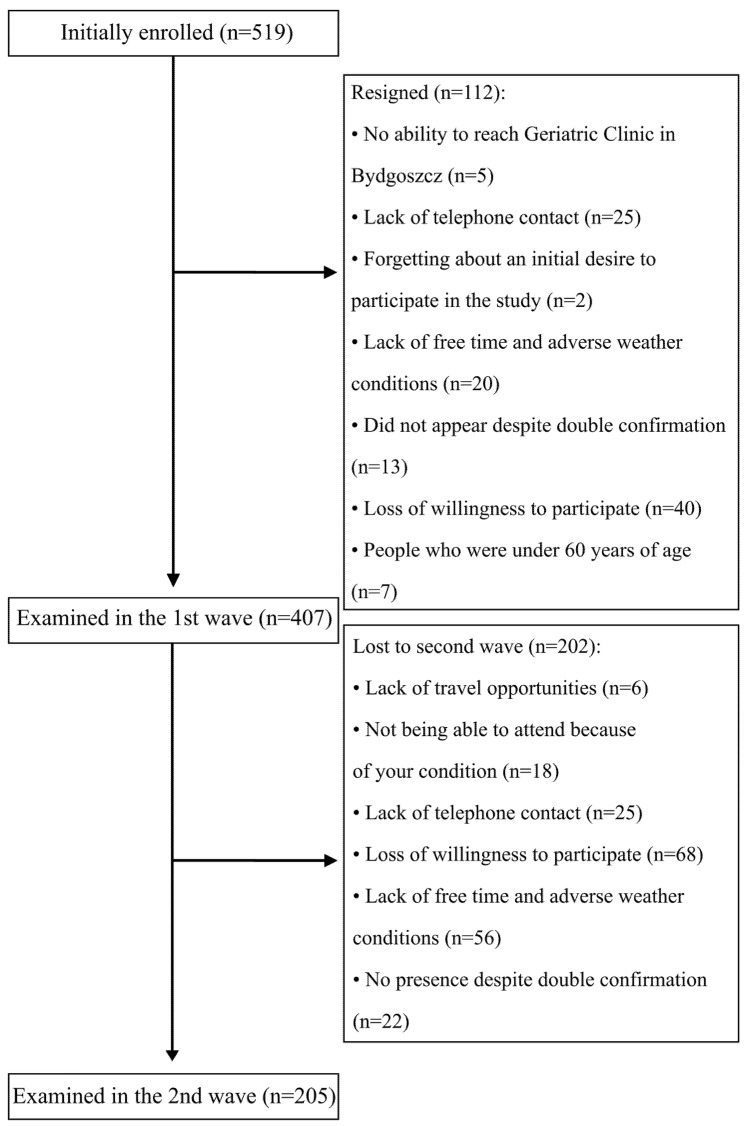
Flow chart of the study.

The study recruitment is summarised in Figure 1. In total, 407 participants (95 males) took part in the baseline series of assessments, with 205 returning two years later for a second assessment (40 males). The comparison between patients re-examined compared to those who were dropouts was described in our previous paper [16].

Recruitment of subjects was done via advertisements on regional television and radio, during the delivery of health-promoting public lectures at Collegium Medicum University, and in meetings of organizations associated with older people in Bydgoszcz, day care centres for the elderly. These advertisements contained information about the opportunity to take part in a free-of-charge examination, including behaviour, body composition, and health state, for people 60 years and over in age. Study volunteers were excluded from participation if they were under 60 years of age. The study was approved by the ethics committee, Ludwik Rydygier Memorial Collegium Medicum in Bydgoszcz, Nicolaus Copernicus University, Torun, Poland (KB 340/2015). Written informed consent was obtained from all participants.

### 2.2. Assessment Methods

#### 2.2.1. Blood Pressure Measurement and Hypertension History Treatment and Assessment

All examinations were completed in a doctor’s office. Heart rate (HR) was assessed by grasping the wrist and measuring via the palpation method, with the variable expressed as beats per minute. Blood pressure was measured on both of the upper limbs in a consecutive manner after at least 5 min of rest. The mean value for each systolic blood pressure (sBP) and diastolic blood pressure (dBP) reading from these two measurements were analysed. The pulse pressure (PP) was calculated using the formula: PP = sBP − dBP. During bassline, as well as the follow up after two years, the patients were asked about drugs taken, including type and dosage. The answers were encoded from market names to names of active compounds. Then, four categories were established: subjects who were not taking antihypertensives drugs in either time points (no to no), which was established as a referring group, and participants who were taking antihypertensive drugs at both time-points (yes to yes). Subjects who were not taking antihypertensive drugs during baseline, but started to take them after two years, were classified as “no to yes”. Participants who were taking antihypertensive drugs during baseline, but ceased taking them after two years, were classified as “yes to no”.

#### 2.2.2. Frequency of Coffee Consumption

Coffee consumption frequency at the follow up time point was examined. Participants were asked to recall the frequency of coffee consumption over the last two years. Answers on questions about frequency of coffee consumption were coded in the following way: “never” was coded as 0, “once a year” as 1, “several times a year” as 2, “1–2 times a month” as 3, “once a week” as 4, “a few times a week” as 5, and “daily” as 6. Then, three categories were distinguished: “never to a few times per month”, “once a week to a few times per week”, and “every day”.

#### 2.2.3. Body Composition Analysis

A Tanita BC-545 body-fat analyser was used to measure weight and body fat both before and after two years. Bioelectric impedance analysis (BIA) was used to measure body composition. Muscle mass was predicted in kilograms and included into the analysis as an alternative to body mass index, which is not an effective body composition predictor [17].

### 2.3. Statistical Analysis

The R package was used to perform statistical analysis [18]. To compare results of tests before vs. after two years, a dependent *t*-test was used for analysis if the assumption was met, otherwise the Wilcoxon test was used. Ggstatsplot package [19] was used to calculate effect sizes and their confidence intervals [−95%; 95%] for dependent comparisons.

Linear mixed models, with restricted maximum likelihood, and the Satterthwaite method, to perform *t*-tests, were carried out using the Lme4 and LmerTest packages [20] to assess the influence of analysed factors on the change of blood pressure (before vs. after two years). Random effects included the subject and time factors, while the rest of the factors included were set as fixed. Both intercepts and slopes were considered as random. The confidence interval (95%) for the coefficient parameters was calculated using the “confint” command to calculate 5000 simulations using the bootstrap method. The “R2beta” command was used to calculate the R^2^ value and its confidence interval (−95%; 95%). A graph illustrating the linear mixed models results was created using the “Dwplot” command.

## 3. Results

The mean age of the participants after the two years follow up assessment was 69.67 (−95% CI = 68.85; 95% CI = 70.5, range 60–88). Table A1 in Appendix A presents the distribution of education level of the re-examined group. Most of participants (84.39%) obtained full secondary education or higher. Eighty-six percent of participants were characterized by a high occupational status (with the highest obtained occupational statuses during lifetime including: a white collar worker, a white collar worker in a managerial position, an owner of craft/entrepreneur, military/policeman/other uniformed services, or seller/employee of trade). Table 1 presents the descriptive statistics of the qualitive predictors included in the model that was used to explain the variance of changes in blood pressure scores after two years.

The fixed predictors included in our model were age at the initial time point (before two years), muscle mass measured after two years, difference in heart rate (HR) in supine position after two years, and the number of hypertensive agents taken between the two time points.

Table 2 show the prevalence of active tobacco smokers between the two time points. Data on the frequency of coffee consumption, sex, and smoking status was included in the linear mixed models. Table A2 presents the time points when the analysed variables were measured.

In the first time point, 101 (49.27%) participants were diagnosed with hypertension during their lifetime. After the two years follow up, this number increased to 107 participants (52.2%), with an average diagnostic age of 59.6 years old (−95% CI = 57.45; 95% CI = 61.75).

**Figure 2:**
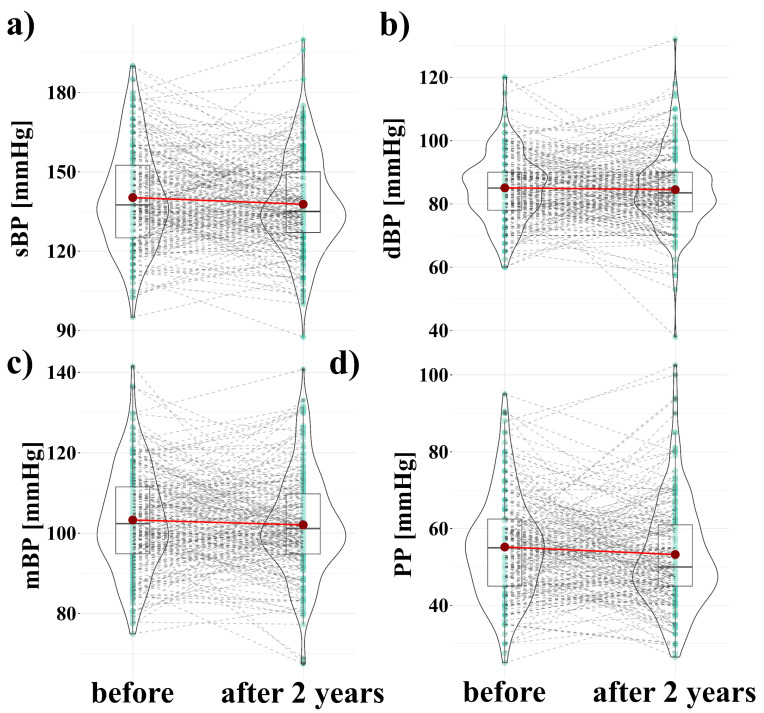
Changes of blood pressure within two years. (**a**) change in systolic blood pressure (sBP) before vs. after 2 years, (**b**) change in diastolic blood pressure (dBP) before vs. after 2 years, (**c**) change in mean blood pressure (mBP) before vs. after 2 years, (**d**) change in pulse pressure (PP) before vs. after 2 years. The shape of the violin graph denotes the distribution of values. A horizontal black line inside the box denotes the median value, while the turquoise dots connected by dashed grey lines indicate the results of individual participants. Dark red dots connected by a light red line indicate the arithmetic mean value.

No statistically significant changes in blood pressure were observed after the two year follow up. The sBP decreased without statistical significance (140.26 ± 19 vs. 137.73 ± 18 mmHg, log_e_(V_Wilcoxon_) = 9.29, *p* = 0.13, r = 0.11 [−0.02; 0.25]) (Figure 2a). No significant changes in dBP were noted (85.06 ± 10.7 vs. 84.49 ± 12.1 mmHg, log_e_(V_Wilcoxon_) = 9.16, *p* = 0.42, r = 0.06 [−0.07; 0.2]) (Figure 2b). No significant changes mBP were noted (103.28 ± 12.5 vs. 102.06 ± 12.7 mmHg, log_e_(V_Wilcoxon_) = 9.33, *p* = 0.25, r = 0.08 [−0.04; 0.22]) (Figure 2c). No significant changes in PP were noted (55.20 ± 13.3 vs. 53.25 ± 14 mmHg, log_e_(V_Wilcoxon_) = 9.36, *p* = 0.06, r = 0.13 [−0.01; 0.25]) (Figure 2d).

Table 3 summarizes the comparison of blood pressure before vs. after two years.

**Figure 3:**
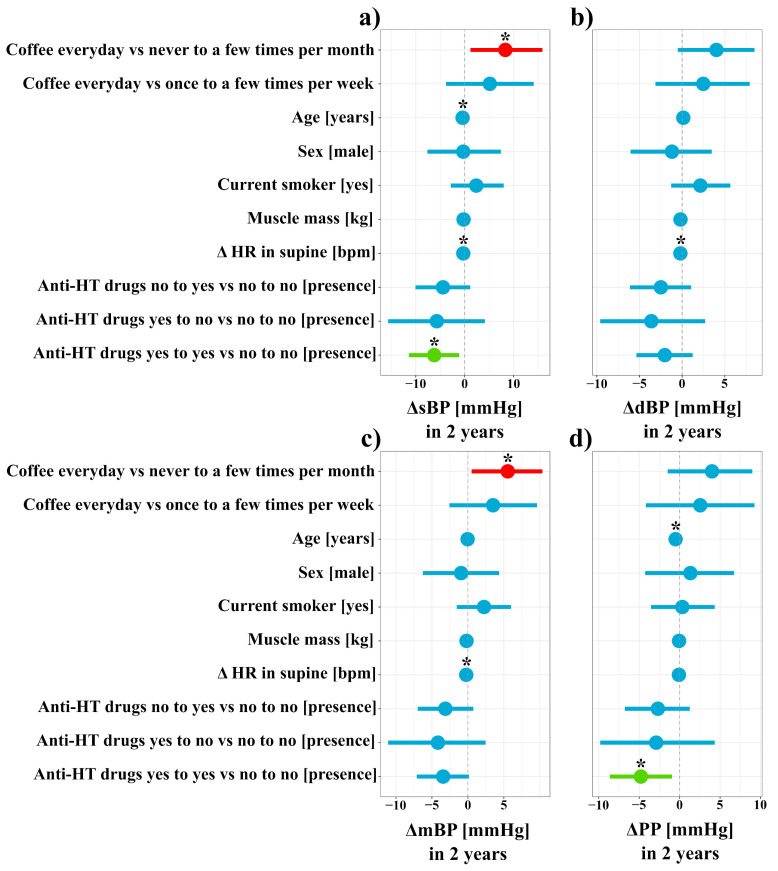
Mixed linear model predicting blood pressure changes. (**a**) change in systolic blood pressure (sBP) before vs. after 2 years, (**b**) change in diastolic blood pressure (dBP) before vs. after 2 years, (**c**) change in mean blood pressure (mBP) before vs. after 2 years, (**d**) change in pulse pressure (PP) before vs. after 2 years. The vertical axis denotes predictors of change in blood pressure, while the horizontal axis illustrates dynamics of blood pressure before vs. after two years. The “0” on the horizontal axis denotes no changes in blood pressure within two years (denoted by a vertical dashed line). Predictors with negative estimates are placed on the left side of the dashed line (factors associated with a decrease in blood pressure after two years). Predictors with positive estimates are placed on the right side of the dashed line. Red horizontal lines illustrate predictors in which both confidence interval values are lower than 0, while blue illustrates predictors in which confidence interval values of estimates cross zero. Green indicate predictors in which both confidence interval values of estimates are greater than 0. “*” mean predictors that are statistically significant (*p* < 0.05). “Δ HR in supine [bpm]” denotes heart rate (HR) in supine position both after two years and before. “Anti-HT drugs no to yes vs. no to no” denotes patients who were not taking antihypertensive drugs during baseline, but started to take after two years in comparison to patients who did not take antihypertensive drugs at either time point. “Anti-HT drugs yes to no vs. no to no” denotes patients who were taking antihypertensive drugs during baseline, but ceased to take them after two years in comparison to patients who did not take antihypertensive drugs at either time point. “Anti-HT drugs yes to yes vs. no to no” denotes patients who were taking antihypertensive drugs during baseline and after two years in comparison to patients who did not take antihypertensive drugs at either time point.

Figure 3a and Table S1 present the results of a mixed linear model with sBP as the predicted variable. The model explained 7% (5%; 15%) of the variance in the change of sBP score before vs. after two years. For every additional year of age, there was a decrease after two years in the sBP of −0.42 mmHg ((−0.78; −0.093), t = −2.27, *p* = 0.02). Participants who drank coffee everyday had a significant increase in sBP compared to participants who drank coffee never or very rarely (never to a few times per month), with a mean of 8.32 mmHg (1.18; 15.91) after two years (t = 2.27, *p* = 0.02). A decrease of HR of 1 bpm after two years was related to a decrease in sBP by −0.28 mmHg (−0.53; −0.02) after 2 years (t = −2.13, *p* = 0.03). Subjects who were taking antihypertensive drugs before and after two years had a decrease in sBP by −6.22 mmHg ((−11.4; −1.11), t = −2.34, *p* = 0.02). The model that predicted changes in mBP explained 6% (5%; 15%) of the variance (Figure 3c and Table S3). Participants who drank coffee everyday had a significant increase in mBP compared to participants who drank coffee never or very rarely (never to a few times per month), with a mean of 5.55 mmHg (0.52; 10.37) after two years (t = 2.17, *p* = 0.03). A decrease of HR by 1 bpm after two years was related to a decrease in mBP by −0.23 mmHg (−0.4; −0.04) after 2 years (t = −2.43, *p* = 0.02). Figure 3d and Table S4 presents the results of the mixed linear model with PP as the predicted variable. The model explained 7% (5%; 16%) of the variance in the change of the PP score before vs. after two years. For every additional year of age, there was a decrease after two years in PP of −0.5 mmHg ((−0.78; −0.22), t = −3.76, *p* = 0.0002). Patients who were taking antihypertensive drugs before and after two years noted a decrease in PP by −4.79 mmHg ((−8.63; −0.93), t = −2.49, *p* = 0.001) in comparison to patients who did take antihypertensive drugs at either time point. The model predicting changes in dBP explained 5% (4%; 13%) of the variance. A decrease of HR by 1 bpm after two years was related to a decrease in mBP by −0.2 mmHg (−0.36; −0.02) after 2 years (t = −2.25, *p* = 0.03) (Figure 3b, Table S2).

## 4. Discussion

### 4.1. Realtionship between Coffee Consumption and Blood Pressure

Our study examined a cohort of older people in Poland, and found that drinking coffee every day was associated with an increase in sBP, with of mean 8.63 mmHg, and mBP, with a mean of 5.85 mmHg, over two years as compared to the subgroup who never or very rarely drank coffee. Moreover, a moderate increase in blood pressure was noted in everyday coffee drinkers in comparison to a subgroup who drank once to a few times per week. The results showed that dBP and PP were not statistically significantly affected by coffee consumption frequency.

An association between a higher intake of coffee consumption and a lower risk of hypertension was noted [12]. In contrast, high coffee consumption was related to high blood pressure [13]. Navarro et al., in longitudinal study on 13.374 participants with a mean age of 35.7 (SD = 10.4), noted that the higher the habitual coffee consumption, the lower the risk of hypertension [21]. According to findings of Navarro et al., authors of a meta-analysis of cohort studies noted a dose-dependent relationship between habitual coffee consumption and the risk of hypertension [22]. Subjects with no coffee intake were compared to those who drank two, four, six, and eight cups of coffee/day, wherein the higher the amount of cups/day of coffee, the lower the risk of hypertension [22]. In a study on 8780 participants (aged 49 (43–55)), it was noted that the relationship between coffee consumption and hypertension occurrence was related to smoking status [23]. Moderate coffee intake (1–3 cups/day) decreased the risk of hypertension in those who had never smoked [23]. De Giuseppe noted that, due to methodological inaccuracies, it is impossible to get a consensus on the relationship between habitual coffee consumption and blood pressure changes [24].

### 4.2. Possible Confounding Factors in Relationship between Coffee Consumption and Blood Pressure

Chlorogenic acid seemed to be a physiologically significant polyphenol with antioxidant properties present in coffee [25]. The total antioxidant activity of coffee depends upon the chemical composition and the degree to which the beans are roasted, with medium-roasted characterised by the highest antioxidant activity [26]. The antioxidant activity of coffee might be different due to method by which the coffee prepared, with espresso characterised as having the highest and decaffeinated coffee as having lower antioxidant activity, respectively. In the current study, no distinction between caffeinated vs. decaffeinated was made, but we assumed that the majority of coffee ingested was of the caffeinated form [27]. Moreover, unfiltered coffee might contain higher amounts of diterpenes, which were related to increasing circulating levels of total cholesterol and in low density lipoprotein form [5]. In addition, the results of recent studies suggested that coffee consumption might alter the gut microbiome [28], and this alternation might be clinically relevant [29]. The authors propose that non-digestible prebiotic included in coffee might mediate this effect, however, there is a requirement for further studies to explore this in more detail [30]. Furthermore, some health benefits from caffeine intake might be mediated via activation of the nuclear factor erythroid 2-related factor-2 system [30]. In addition, the effects of coffee consumption might be modified by genetic factors. Participants with higher genetic risk were characterised by high BP in relation to higher coffee consumption [13]. In contrast to that observation, a recent study was unable to confirm that genetic variance related to caffeine metabolism affect in the relationship between coffee consumption and cardiovascular disorder risk [31]. Therefore, further studies on coffee consumption should utilise a longitudinal approach with deep phenotyping methods to assess different pharmacologically active ingredients of coffee on human physiology on multi-level dimensions.

### 4.3. Physiological Differences between Systolic and Diastolic Blood Pressure

In our current study, the frequency of coffee consumption was related to sBP, but not dBP. As discussed above, isolated systolic hypertension might be a result of age-associated changes in the cardiovascular system [2]. Pulse pressure is one of the indicators of aortic stiffness [32].

### 4.4. Study Limitations

In the current study, only self-reported recall of the frequency of coffee consumption was measured, without an indication of the quantity nor quality. Such methods should be incorporated in future longitudinal studies conducted in Poland. As mentioned, the method of coffee preparation has an influence on the quality and quantity of substances introduced upon consumption [5,33,34]. It was not possible to analyse the method of coffee preparation in the currently analysed sample. It was reported that 45% consumed coffee made with powder, while 40% consumed instant coffee as the most common form in Poland [35]. Based on the questionnaire results from 398 responders in Poland, 48% drink espresso prepared under pressure in a machine. In total, 42% preferred pouring hot water over coffee (coffee in a glass or cup, without filtering, with coffee grounds) [36]. Therefore, arguably it might be predicted that the vast majority of coffee prepared by the participants in the current sample was prepared using those two methods. In our previous study, we highlighted a need for further longitudinal studies and the incorporation of a more representative sample with a higher proportion of males [16]. The results of previous studies indicated between-sex differences in the health effects of phenolic compounds [37]. Older females were characterised by better tolerability of frailty related to aging [38]. In addition, many between-sex differences in the trajectory of inflammation, metabolism, and allostatic load were noted [39]. Between-sex differences in the health effects of habitual coffee drinking were noted in a meta-analysis of prospective trials, where lower risk of coronary heart disease was noted in a group of female habitual coffee drinkers [40]. In addition, participants who regularly drink coffee might also be characterised by different patterns of behaviour. Mattioli et al. noted a good adherence to the Mediterranean diet and high levels of physical activity in a group of women with high coffee consumption [34]. In the current study, the pattern of diet and physical activity was not evaluated in an objective manner, therefore the current results could not be adjusted by those factors. Results of the current study should be adjusted not only by the presence, or absence, of antihypertension drug treatment, but also by the precise hypertension duration, specific antihypertensive drug type, its quantity, and dosage. In addition, the method of recruitment into study via mass media could have led to bias, as well as high dropout rate, and should also be considered a limitation [16].

## 5. Conclusions

In this study we found, in a cohort of older people in Poland, that the daily consumption of coffee was related to an increase in sBP and in mBP over two years when compared to a subgroup who never or very rarely drank coffee. Moreover, the increase in blood pressure in daily coffee drinkers, compared to a subgroup who drink once to a few times per week, was noted, however, it was not statistically significant. DBP and PP were not affected by coffee consumption frequency in a statistically significant manner.

## Figures and Tables

**Table 1:** Quantitative variables included in linear mixed model.

Variables	Mean ± SD
Age before (years)	69.67 ± 6
Muscle mass before (kg)	45.54 ± 8.2
Muscle mass after two years (kg)	44.87 ± 6.7
HR before (bpm)	67.57 ± 8
HR after (bpm)	69.6 ± 9.8

HR—heart rate.

**Table 2:** Qualitative variables included in linear mixed model.

Parameter	Level of Estimate	Number	%
Sex	Male	40	19.5
	Female	165	80.5
Current smoker	No	165	80.5
	Yes	38	18.5
	Missing data	2	1
Anti-HT drugs	yes to no	10	4.9
	yes to yes	62	30.2
	no to yes	49	23.9
	no to no	84	41
Coffee consumption frequency	never to a few times per month	19	9.3
	once a week to a few times per week	26	12.7
	every day	160	78

**Table 3:** Comparison blood pressure before vs. after two years.

Variable	Mean ± SD before	Mean ± SD after 2 Years	log_e_(V_Wilcoxon_)	*p*-Value
sBP	140.26 ± 19	137.73 ± 18	9.29	0.13
dBP	85.06 ± 10.7	84.49 ± 12.1	9.16	0.42
mBP	103.28 ± 12.5	102.06 ± 12.7	9.33	0.25
PP	55.20 ± 13.3	53.25 ± 14	9.36	0.06

sBP—systolic blood pressure, dBP—diastolic blood pressure, mBP—mean blood pressure, PP—pulse pressure.

## Data Availability

Individual data are available from the corresponding author S.K. upon request.

## Supplementary Materials

The following are available online at https://www.mdpi.com/article/10.3390/nu13103372/s1, Table S1: Mixed linear model predicting systolic blood pressure changes, Table S2: Mixed linear model predicting diastolic blood pressure changes, Table S3: Mixed linear model predicting mean blood pressure changes., Table S4: Mixed linear model predicting pulse pressure changes.

## Appendix A

**Table A1:** Distribution of education categories in the re-examined group.

Category	Count	Percent
Primary education (incomplete)	1	0.49
Primary education	3	1.46
Vocational school education	22	10.73
Secondary education (incomplete)	6	2.93
Secondary education	92	44.88
Higher professional/Engineering education	26	12.68
Master’s degree	52	25.37
PhD degree and higher	3	1.46

**Table A2:** Time points at which the variables included in linear mixed model were measured.

Variables	Time Points at Which the Variables Were Measured
Coffee consumption	after
Age (years)	before
Sex	before
Current smoker	after
Muscle mass before (kg)	before and after two years
Anti-HT drugs (quantity)	before and after two years
Δ in HR (bpm)	before and after two years
