# Supplementary material for: Coffee Consumption and Blood Pressure: Results of the Second Wave of the Cognition of Older People, Education, Recreational Activities, Nutrition, Comorbidities, and Functional Capacity Studies (COPERNICUS)
Source: Nutrients. 2021 Sep 25;13(10):3372. doi: 10.3390/nu13103372 (PMC8538539; doi:10.3390/nu13103372)
Supplement: Supplementary file 1 [file nutrients-13-03372-s001.zip › nutrients-1352457-supplementary.pdf]

Table S1. Mixed linear model predicting systolic blood pressure changes.

Variable	Estimate	-95% CI	95% CI	T-statistic	*p*-value
Coffee everyday vs never to a few times per month	8.32	1.18	15.91	2.27	0.02
Coffee everyday vs once to a few times per week	5.15	-3.80	14.13	1.15	0.25
Age [years]	-0.42	-0.78	-0.03	-2.27	0.02
Sex [male]	-0.28	-7.62	7.41	-0.07	0.94
Current smoker [yes]	2.38	-2.86	7.99	0.87	0.39
Muscle mass [kg]	-0.22	-0.61	0.15	-1.15	0.25
Δ HR in supine [bpm]	-0.28	-0.53	-0.02	-2.13	0.03
Anti-HT drugs no to yes vs no to no [presence]	-4.43	-10.06	1.14	-1.55	0.12
Anti-HT drugs yes to no vs no to no [presence]	-5.70	-15.66	4.14	-1.15	0.25
Anti-HT drugs yes to yes vs no to no [presence]	-6.22	-11.40	-1.11	-2.34	0.02

Table S2. Mixed linear model predicting diastolic blood pressure changes.

Variable	Estimate	-95% CI	95% CI	T-statistic	*p*-value
Coffee everyday vs never to a few times per month	4.02	-0.51	8.49	1.74	0.08
Coffee everyday vs once to a few times per week	2.48	-3.13	7.93	0.88	0.38
Age [years]	0.14	-0.08	0.37	1.21	0.23
Sex [male]	-1.19	-6.05	3.48	-0.48	0.63
Current smoker [yes]	2.14	-1.28	5.65	1.23	0.22
Muscle mass [kg]	-0.19	-0.44	0.06	-1.54	0.13
Δ HR in supine [bpm]	-0.20	-0.36	-0.02	-2.25	0.03
Anti-HT drugs no to yes vs no to no [presence]	-2.49	-6.11	1.06	-1.37	0.17
Anti-HT drugs yes to no vs no to no [presence]	-3.61	-9.60	2.71	-1.15	0.25
Anti-HT drugs yes to yes vs no to no [presence]	-2.03	-5.36	1.24	-1.20	0.23

Table S3. Mixed linear model predicting mean blood pressure changes.

Variable	Estimate	-95% CI	95% CI	T-statistic	*p*-value
Coffee everyday vs never to a few times per month	4.02	-0.51	8.49	1.74	0.08
Coffee everyday vs once to a few times per week	2.48	-3.13	7.93	0.88	0.38
Age [years]	0.14	-0.08	0.37	1.21	0.23
Sex [male]	-1.19	-6.05	3.48	-0.48	0.63
Current smoker [yes]	2.14	-1.28	5.65	1.23	0.22
Muscle mass [kg]	-0.19	-0.44	0.06	-1.54	0.13
Δ HR in supine [bpm]	-0.20	-0.36	-0.02	-2.25	0.03
Anti-HT drugs no to yes vs no to no [presence]	-2.49	-6.11	1.06	-1.37	0.17
Anti-HT drugs yes to no vs no to no [presence]	-3.61	-9.60	2.71	-1.15	0.25
Anti-HT drugs yes to yes vs no to no [presence]	-2.03	-5.36	1.24	-1.20	0.23

Table S4. Mixed linear model predicting pulse pressure changes.

Variable	Estimate	-95% CI	95% CI	T-statistic	*p*-value
Coffee everyday vs never to a few times per month	4.00	-1.48	8.99	1.52	0.13
Coffee everyday vs once to a few times per week	2.55	-4.18	9.26	0.79	0.43
Age [years]	-0.50	-0.78	-0.22	-3.76	0.0002
Sex [male]	1.33	-4.27	6.74	0.48	0.64
Current smoker [yes]	0.33	-3.53	4.33	0.17	0.87
Muscle mass [kg]	-0.07	-0.36	0.21	-0.51	0.61
Δ HR in supine [bpm]	-0.09	-0.28	0.12	-0.85	0.40
Anti-HT drugs no to yes vs no to no [presence]	-2.69	-6.77	1.26	-1.30	0.20
Anti-HT drugs yes to no vs no to no [presence]	-2.90	-9.82	4.34	-0.81	0.42
Anti-HT drugs yes to yes vs no to no [presence]	-4.79	-8.63	-0.93	-2.49	0.01
